# Platelet-Mediated Metabolism of the Common Dietary Flavonoid, Quercetin

**DOI:** 10.1371/journal.pone.0009673

**Published:** 2010-03-12

**Authors:** Bernice Wright, Trevor Gibson, Jeremy Spencer, Julie A. Lovegrove, Jonathan M. Gibbins

**Affiliations:** 1 Institute for Cardiovascular and Metabolic Research, School of Biological Sciences, University of Reading, Reading, United Kingdom; 2 Biocentre Facility, University of Reading, Reading, United Kingdom; 3 School of Food and Nutritional Sciences, University of Reading, Reading, United Kingdom; 4 Blood Transfusion Research Group, King Saud University, Riyadh, Saudi Arabia; Auburn University, United States of America

## Abstract

**Background:**

Flavonoid metabolites remain in blood for periods of time potentially long enough to allow interactions with cellular components of this tissue. It is well-established that flavonoids are metabolised within the intestine and liver into methylated, sulphated and glucuronidated counterparts, which inhibit platelet function.

**Methodology/Principal Findings:**

We demonstrate evidence suggesting platelets which contain metabolic enzymes, as an alternative location for flavonoid metabolism. Quercetin and a plasma metabolite of this compound, 4′-*O*-methyl quercetin (tamarixetin) were shown to gain access to the cytosolic compartment of platelets, using confocal microscopy. High performance liquid chromatography (HPLC) and mass spectrometry (MS) showed that quercetin was transformed into a compound with a mass identical to tamarixetin, suggesting that the flavonoid was methylated by catechol-*O*-methyl transferase (COMT) within platelets.

**Conclusions/Significance:**

Platelets potentially mediate a third phase of flavonoid metabolism, which may impact on the regulation of the function of these cells by metabolites of these dietary compounds.

## Introduction

**Figure 1:**
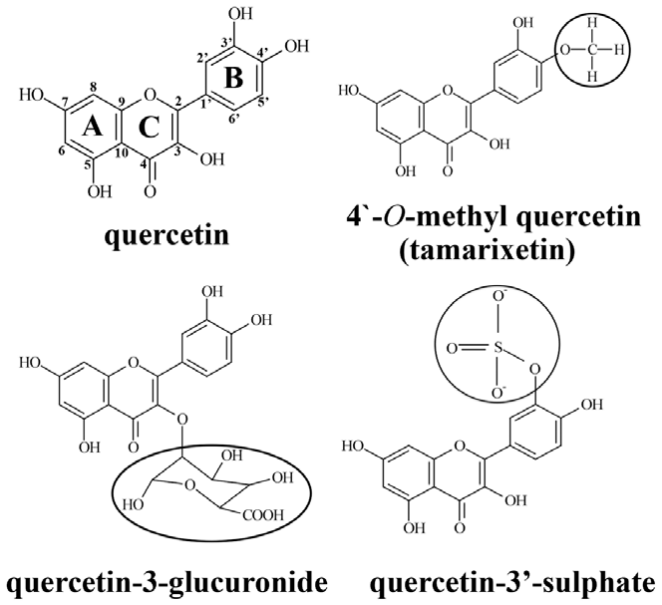
The structures of quercetin aglycone and plasma metabolites. Quercetin is part of the flavonol subclass of flavonoids. Metabolites of quercetin include methylated (4′-*O*-methyl quercetin: tamarixetin), sulphated (quercetin-3′-sulphate) and glucuronidated (quercetin-3-glucuronide) counterparts.

Following the ingestion of fruits and vegetables, flavonoids (Figure 1) abundantly present in these dietary sources are metabolised [1]. First pass metabolism in enterocytes lining the wall of the small intestine and subsequent transformations within liver hepatocytes generate *O*-methylated, glucuronidated and sulphated metabolites (Figure 1) [2]–[9]. It is possible that flavonoid metabolism is a multistep process that occurs in other locations including their main transport system, blood. Plasma transit times of flavonoid metabolites of 30 min-11 h [10], [11] may allow these compounds sufficient time to interact with cellular components of blood. The plasma metabolite of quercetin, 4′-*O*-methyl quercetin (tamarixetin), has been reported to be taken up by erythrocytes [12], [13], and the *ex vivo* inhibition of platelet-leukocyte associations following ingestion of cocoa flavonoids [14] suggest interactions of the metabolites of these compounds with leukocytes.

As both erythrocytes [15], [16] and leukocytes [17] contain COMT, flavonoid metabolites may undergo further metabolism within these cells. The structures of these compounds may also be modified by platelets equipped with enzymes capable of modifying molecules through addition of a methyl (COMT) [18], [19], a sulphate (phenol sulphotransferases P-ST or SULT1A1) [20] or a glucuronide (glucuronosyltransferases) [21] group. Glucuronide or sulphate groups conjugated to flavonoids within platelets would deter transport of these compounds across the membrane, allowing inhibition of platelet signalling by metabolites. Modification of these compounds by metabolic enzymes within blood cells including platelets have not, however, been examined. Platelets play a central role in haemostasis [22], [23] through their involvement in the repair of minor vascular injuries, and they also mediate the pathophysiological process, thrombosis [24], [25], when their unregulated activation leads to the formation of aggregates which block arteries. These cells also represent targets for the physiological actions of flavonoid metabolites [10], [26]. In the present study, we have investigated the metabolic capabilities of platelets by studying their ability to transform the structures of the common dietary flavonoid, quercetin and a plasma metabolite of this flavonol, tamarixetin.

## Results

### Quercetin and tamarixetin are internalised by platelets

**Figure 2:**
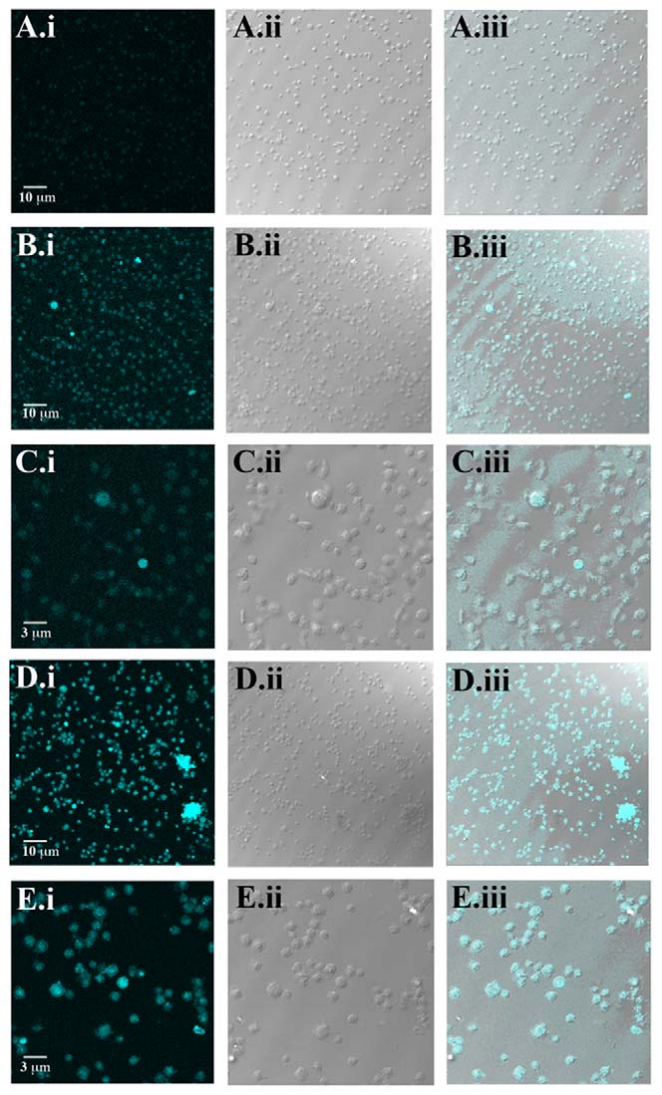
Platelets internalise quercetin and tamarixetin. Platelets suspended to a density of 2×10^8^ cells.mL^−1^ were incubated with quercetin (100 µM: (B.i-iii) and (C.i-iii)), tamarixetin (40 µM: (D.i-iii) and (E.i-iii)) or solvent control (DMSO (0.2% (v/v)): (A.i-iii)) for 30 min. Fluorescence was detected at 480 nm–500 nm after excitation at 430 nm with an argon laser. Images of a single middle layer from z-stacks are shown (DMSO control: (A.i-iii), quercetin: (B.i-iii) tamarixetin: (C.i-iii)) and higher magnifications of areas of interest are also shown (quercetin: (D.i-iii), tamarixetin: (E.i-iii)). Images represent results from at least 3 individual experiments.

To determine whether quercetin and tamarixetin were able to gain access to the platelet cytosol containing metabolic enzymes, the intrinsic fluorescent properties of quercetin and tamarixetin [27] were utilised to visualise their potential presence within these cells. Following incubation with quercetin (100 µM), tamarixetin (40 µM) or the solvent control, dimethylsulphoxide (DMSO: 0.2% (v/v)) for 30 min, a series of images in the z dimension were obtained at 0.2 µm intervals through the membrane and cytosol of platelets. A single image of a layer from the middle of the series illustrated internalisation of quercetin (Figure 2B. i-iii and 2C. i-iii) and tamarixetin (Figure 2D. i-iii and 2E. i-iii) and magnified images showed greater detail (quercetin: Figure 2C. i-iii) and (tamarixetin: Figure 2E. i-iii). Low levels of auto-fluorescence were detected in untreated platelets (Figure 2A.i-iii).

### Quercetin and tamarixetin associate with platelets

**Figure 3:**
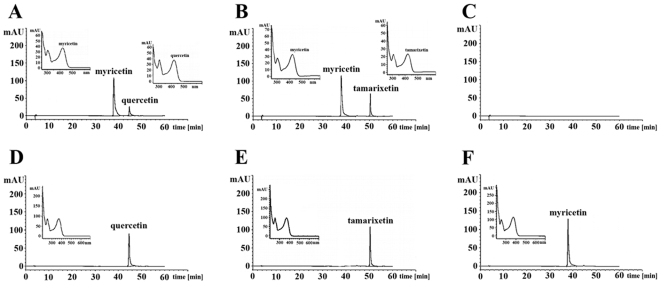
Quercetin and tamarixetin associate with platelets. Quercetin (50 µM) and tamarixetin (50 µM) were incubated with platelets (8×10^8^ cells.mL^−1^) for 5, 40, 60 or 120 min prior to lysis with 50% (v/v) methanol and 0.1% (v/v) HCl. Compounds within extracts obtained from platelet lysates spiked with myricetin (external control compound of similar structure) were separated over 60 min using HPLC analysis with photodiode array detection. Chromatograms show quercetin ((A)-RT: 46.1 min) and tamarixetin ((B)-RT: 51.6 min) associated with platelets through comparison with standards (quercetin: D; tamarixetin: 3E), untreated platelets (plasma control: C) and the external control, myricetin ((A, B)-RT: 39 min; F: standard). Insets show UV absorbance spectrum (λ: 360 nm) of detected compounds. Data represent 3 individual experiments.

The putative ability of platelets to metabolise quercetin and tamarixetin was investigated by HPLC analysis which involved measuring the presence of these compohunds and potential metabolic products in extracts from platelets treated with the flavonol and metabolite. Platelets were treated with quercetin (50 µM), tamarixetin (50 µM), or DMSO (0.2% (v/v)) for 5, 40, 60 and 120 min, lysed and spiked with myricetin (50 µM). Quercetin and tamarixetin were identified at all incubation periods in platelet extracts (through comparison with appropriate standards), but were not modified. The retention times (RT) of quercetin (Figure 3A) and tamarixetin (Figure 3B) were 46.1 and 51.6 min respectively and that of the external control spike, myricetin (Figure 3F) was 39 min. The plasma control (Figure 3C) did not contain any compounds and HPLC analysis of standards showed single peaks at the respective RT stated above (Figure 3D, E and F). UV spectra of quercetin, myricetin and tamarixetin measured at 360 nm confirmed the presence of these compounds in platelet extracts.

### Quercetin is metabolised by platelets

**Figure 4:**
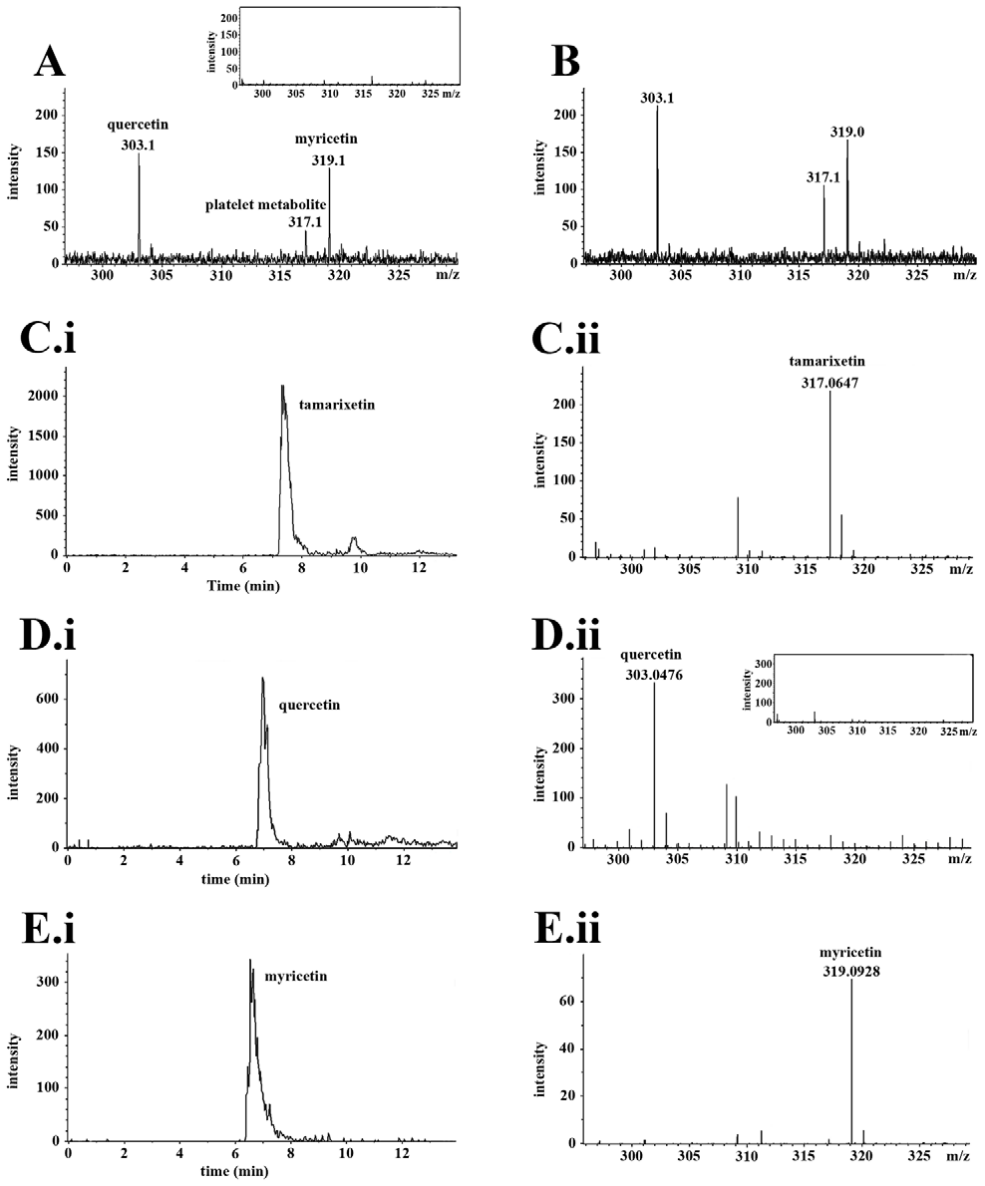
Quercetin is metabolised by platelets. Platelets (8×10^8^ cells.mL^−1^) pretreated with quercetin (50 µM) and tamarixetin (50 µM) for 5, 40, 60 or 120 min were lysed with 50% methanol and 0.1% formic acid. Extracts from lysed platelets were separated by HPLC utilising UV detection (λ: 210 nm) over a period of 20 min, before identification of the protonated masses of compounds using mass spectrometry. Mass spectrums show the ([M+H^+^]^+^) of quercetin (molecular mass: 303 Da), the platelet metabolite (molecular mass: 317 Da) and the external control spike, myricetin (molecular mass: 319 Da) at 5 min (A: with plasma control inset) and 60 min (B). EIC and mass spectrums of standards show tamarixetin (EIC: C.i-RT: 7.5 min; mass spectrum: C.ii-molecular mass: 317 Da), quercetin (EIC: (D.i)-RT: 7 min; mass spectrum: (D.ii)-molecular mass: 303 Da) with inset MS spectrum showing the absence of tamarixetin (ii) and myricetin (EIC: (E.i)-RT: 6.5 min; mass spectrum: (E.ii)-molecular mass: 319 Da). Data represent 3 individual experiments using platelets isolated from 3 different blood donors.

Internalisation of quercetin and tamarixetin by platelets raised the possibility that the flavonol and metabolite may be metabolised by COMT, P-ST or glucuronidases within these cells. The concentration and analysis of platelet extracts by HPLC alone was insufficiently sensitive to detect metabolites of compounds, so MS was incorporated following HPLC separation. Platelets were incubated for 5, 40, 60 and 120 min with quercetin (50 µM) or tamarixetin (50 µM), before lysis and extraction. Quercetin aglycone (Figure 4A) was converted into a compound that was detected at an RT (7.5 min) and m/z (317) identical to those of tamarixetin. The platelet metabolite was detected at low intensity between 5 (Figure 4A) and 40 min (data not shown) with the ion detected at higher intensity after 60 min, suggesting a higher internal concentration (Figure 4B). This species was not detected at 0 and 120 min. Tamarixetin was not modified by platelets (data not shown). Other plasma metabolites of quercetin, quercetin-3′-sulphate and quercetin-3-glucuronide were not detected within platelets treated with either the aglycone or methylated metabolite (data not shown).

The intensity of the platelet metabolite and quercetin (RT: 7 min; m/z: 303) increased together (Figure 4A and 4B) possibly due to increased uptake of quercetin over 5–60 min, and the intensity of the external control spike, myricetin (RT: 6.5 min; m/z: 319) remained unmodified (compare Figure 4A to Figure 4B). To determine that the detected metabolite was due to modification of quercetin by platelets, untreated platelets (4A inset: plasma control) and quercetin aglycone (4D.ii inset) were examined to confirm the absence of tamarixetin. The plasma control extracted ion chromatogram (EIC) was gated at the RT range for quercetin, tamarixetin and myricetin (4A inset), and the quercetin standard EIC was gated at the RT for tamarixetin (4D.ii inset). Very low levels of erythrocytes (<3% of total cell volume) and leukocytes (<0.02% of total cell volume) which contain COMT [15]–[17] were present within platelet preparations, so it is unlikely, that these cells were responsible for metabolism of quercetin. Taken together, these data indicated that quercetin may be methylated at the B ring C-4′ position within platelets.

## Discussion

The modification of flavonoid metabolites by platelets circulating in blood is possible as these cells contain metabolic enzymes [18]–[21]. A clear understanding of the metabolism of flavonoids within plasma may provide greater insight into their pharmacokinetics. It has been reported that quercetin metabolites are retained within plasma for periods of time ranging from 30 min - 11 h [10], [11], and these slow rates of excretion together with bioavailability profiles of metabolites of this flavonol (100 mg of quercetin from onions or quercetin-4′-glucoside - 7-7.6 µM quercetin in plasma (11)) suggest they may accumulate with repeated dietary intake. Therefore, inhibition of signalling proteins by quercetin metabolites may be due to the concentration of their levels within cells.

The present study is the first to demonstrate flavonoid internalisation and metabolism by platelets. Flavonoids and metabolites have been shown to be taken up by erythrocytes [12], [13], but the structures of these compounds were not reported to be modified within these cells. Other cells, however, including dermal fibroblasts [28], neural astrocytes and microglia [29], intestinal enterocytes [2]–[6] and liver hepatocytes [7]–[9] have been shown to generate methylated, sulphated and glucuronidated metabolites of flavonoids.

Quercetin was transformed into a compound with a similar mass as a plasma metabolite of the flavonol, 4′-*O*-methyl quercetin (tamarixetin), indicating methylation of the B ring catechol group (see Figure 1). Methylation of quercetin by platelets is feasible as these cells contain COMT [18], [19]. Although bioavailability profiles observed following the ingestion of quercetin supplements [10] or dietary sources [11], [26] indicate that quercetin aglycone is not present physiologically, modification of the structure of this flavonoid by platelets suggests that more extensive metabolism of quercetin metabolites may occur within these cells. Therefore, the inhibition of platelet function by quercetin may occur if this compound was produced as an intermediate during the further metabolism of internalised metabolites by these cells. The 4′-*O*-methylated metabolite, tamarixetin, was not altered, but the ability of this compound to gain access to the platelet cytosolic compartment (Figure 3) demonstrated the potential for modification of the structure of this metabolite through the addition of sulphate or glucuronide groups on the A–C ring complex (see Figure 1).

In the present study, a 2-fold increase in the intensity of the platelet metabolite ion between 5 and 60 min (compare Figure 4A and 4B) was observed, but the quercetin ion was detected at high intensity at all incubation periods. These changes in intensity were suggestive of similar changes in levels of compounds. Typically, studies investigating flavonoid metabolism demonstrate a reduction in levels of the compound undergoing metabolism concurrently with an increase in levels of the metabolic product. Previously reported metabolism of quercetin by dermal fibroblasts demonstrated increasing levels of the flavonol for up to 6 h whilst levels of generated metabolites increased from 2–18 h [28], and in neural cells levels of quercetin declined steadily over a period of 12 h as metabolite levels increased for 18 h [29]. Loss of the metabolic product after 120 min, potentially indicates degradation or externalisation of this compound after 60 min. The production and export of methylated quercetin metabolites (isorhamnetin and tamarixetin) by neural cells has been reported [29].

We have demonstrated that platelets are capable of metabolising quercetin potentially through methylation. As flavonoid metabolism within the gut (Phase I) and liver (phase II) is well-established, this finding suggests that the negative modulation of platelet function by flavonoid metabolites [10], [26] may be mediated by these compounds generated within these cells and after they are internalised. Quercetin metabolite inhibitory mechanisms for platelet function may include antagonism of surface receptors; they may bind to the thromboxane A_2_ receptor, as the parent flavonoid has been demonstrated to bind to this receptor [30], [31]. We conclude that a third phase of flavonoid metabolism involving platelets and other blood cells containing metabolic enzymes may occur *in vivo*.

## Materials and Methods

### Ethics Statement

Blood was obtained from healthy aspirin-free human volunteers with written informed consent, following approval from the University of Reading Research Ethics Committee.

### Materials

Quercetin, tamarixetin and myricetin were purchased from Extrasynthese (Genay, France) and solubilised in DMSO from Sigma (Poole, UK). Acetonitrile, formic acid, prostacyclin (PGI_2_), sodium chloride (NaCl), hydrated disodium hydrogen phosphate (Na_2_HPO_4_.12H_2_O) and magnesium chloride (MgCl_2_) were also obtained from Sigma. Quercetin-3′-sulphate was prepared as previously described [32] and queretin-3-glucuronide was purified from French beans by preparative HPLC. Hydrochloric acid (HCl) and methanol were from Fisher Scientific (Leicestershire, UK). Potassium chloride (KCl) and sodium hydrogen carbonate (NaHCO_3_) were bought from Fisons Plc (Loughborough, UK), and VectorShield® was from Molecular Probes (Invitrogen Ltd.; Paisley, UK).

### Analysis of flavonoid and metabolite internalisation

Washed platelets isolated as described previously [33] and suspended to a density of 2×10^8^ cells.mL^−1^ in modified Tyrode's-HEPES (134 mM NaCl, 0.34 mM Na_2_HPO_4_, 2.9 mM KCl, 12 mM NaHCO_3_, 20 mM HEPES, 1 mM MgCl_2_, pH 7.3) buffer, were incubated with quercetin (100 µM), tamarixetin (40 µM) or DMSO (0.2% (v/v)) for 30 min. Cells were centrifuged at 1400×*g*, the supernatant was removed and pellets were washed twice with Tyrode's-HEPES buffer before fixation in 3.7% paraformaldehyde for 20 min. To preserve the intrinsic fluorescence of quercetin and tamarixetin, fixed cells were treated with VectorShield® prior to mounting for confocal microscopy analysis. Fluorescence was excited at 430 nm with an argon laser and emitted at 480 nm–500 nm. Three dimensional representations of platelets were constructed by the generation of sections in the z dimension and compiled into z-stacks.

### HPLC and MS analyses

Platelets isolated as described previously [33] were suspended to a density of 8×10^8^ cells.mL^−1^ before treatment with quercetin (50 µM), tamarixetin (50 µM), myricetin (50 µM) or DMSO (0.2% (v/v)) for 5, 40, 60 and 120 min, in the presence of 0.05 µg.mL^−1^ PGI_2_, to prevent activation. Treated platelets were centrifuged at 1400×*g* for 10 min at room temperature to remove excess compound and resuspended with 0.05 µg.mL^−1^ PGI_2_ in modified Tyrode's-HEPES buffer. Platelets and compounds were incubated at 30°C for 30 min and a final centrifugation step (1400×*g* for 10 min) was performed to remove residual compound. Platelet pellets were lysed rapidly on ice in aqueous methanol (50% (v/v)) containing 0.1% HCl and left to solubilise for 30 min. For samples which underwent LC prior to MS analysis, 0.1% formic acid was utilised instead of 0.1% HCl to allow positive ion mode analyses to be conducted. The lysate was centrifuged (1400×*g* for 10 min at 4°C) and the supernatant/extract was retained for LC and MS analyses. Platelet extracts were spiked with 50 µM myricetin, an internal control compound of similar structure.

Reversed phase HPLC analyses used to demonstrate flavonoid association with platelets were conducted using an Agilent 1100 LC system containing a Novapak C18 column. The total elution time for compounds detected using a photodiode array and UV detection (max. absorbance: 360 nm) was 60 min. HPLC separation performed prior to MS analysis were conducted using a gradient increasing buffer B (5% H_2_O, 94.9% acetonitrile and 0.1% formic acid) from 5% to 100% (and decreasing buffer A (99.9% H_2_O and 0.1% formic acid)) within 20 min. Eluted species were detected by UV spectroscopy (λ: 210 nm). Appropriate flavonoid and metabolite standards were used to identify compounds, and the LC/MS system was calibrated externally using agilent tune mix (G2421A) that generated ions at m/z 118, 322, 622 and 922. A time of flight (TOF) mass spectrometer (Microtof) was utilised to determine masses of compounds. The molecular masses of quercetin and metabolites are between 300 and 500 daltons (Da), so a mass/charge (m/z) scan range of 100–1100 Da was used, and the instrument was calibrated in positive ion mode as extracted compounds were protonated. The Data Analysis (DA) software package (Bruker, Daltonics) was utilised to isolate masses and chromatograms of individual compounds, and masses were isolated from total ion chromatograms (TIC) by gating the retention time (RT) of individual compounds within an EIC.
